# Antibacterial Activity of an Anodized TiNbSn Alloy Prepared in Sodium Tartrate Electrolyte

**DOI:** 10.3389/fbioe.2022.883335

**Published:** 2022-04-11

**Authors:** Hiroaki Kurishima, Yu Mori, Keiko Ishii, Hiroyuki Inoue, Takayuki Mokudai, Satoko Fujimori, Eiji Itoi, Shuji Hanada, Naoya Masahashi, Toshimi Aizawa

**Affiliations:** ^1^ Department of Orthopedic Surgery, Tohoku University Graduate School of Medicine, Sendai, Japan; ^2^ Department of Medical Microbiology, Mycology and Immunology, Tohoku University Graduate School of Medicine, Sendai, Japan; ^3^ Department of Materials Science, Graduate School of Engineering, Osaka Prefecture University, Sakai, Japan; ^4^ Institute for Materials Research, Tohoku University, Sendai, Japan

**Keywords:** antibacterial activity, anodic oxide, photocatalyst (TiO_2_), sodium tartrate, TiNbSn alloys

## Abstract

In this study, we anodized a TiNbSn alloy with low Young’s modulus in an electrolyte of sodium tartrate with and without hydrogen peroxide (H_2_O_2_). The photo-induced characteristics of the anodized alloy were analyzed for crystallinity and electrochemical conditions with comparisons to the effect with the addition of H_2_O_2_. The antibacterial activity was evaluated using methicillin-resistant *Staphylococcus aureus* and other pathogenic bacteria according to ISO 27447, and time decay antibacterial tests were also conducted. The anodized oxide had a porous microstructure with anatase- and rutile-structured titanium dioxide (TiO_2_). In contrast, the peaks of rutile-structured TiO_2_ were accelerated in the anodized TiNbSn alloy with H_2_O_2_. The formation of hydroxyl radicals and methylene blue breaching performance under ultraviolet irradiation was confirmed in the anodic oxide on TiNbSn alloy with and without H_2_O_2_. The anodic oxide on TiNbSn alloy had a robust antibacterial activity, and no significant difference was detected with or without H_2_O_2_. We conclude that anodized TiNbSn alloy with sodium tartrate electrolyte may be a functional biomaterial with a low Young’s modulus and an antibacterial function.

## Introduction

The development of antibacterial metals has been in use with antibacterial metal ions such as silver and copper, or antibacterial agents such as iodine and vancomycin (Ando et al., 2010; Shirai et al., 2011; Han et al., 2017; Wang et al., 2019). However, there is a risk that these added toxic substances have the potential to leak into the bloodstream and result in subsequent side effects (Punjataewakupt et al., 2019). Titanium dioxide (TiO_2_) has previously been reported as a photocatalytic material and is used for air and water purification due to decomposition of the redox species by photogenerated carriers upon illumination corresponding to bandgap energy (Fujishima and Honda, 1972). In the medical field, there are reports that TiO_2_ also has an antibacterial activity (Ohko et al., 2001; Yao et al., 2008) and an antitumor effect (Cai et al., 1992; Kubota et al., 1994). The irradiation of water on TiO_2_ by ultraviolet (UV) light results in its decomposition by the photocatalytic effect to produce reactive oxygen radicals (ROS) such as hydroxyl radicals (•OH), superoxide anions, and hydrogen peroxide (H_2_O_2_) (Fujishima and Honda, 1972). The ROS has been reported to have antibacterial and antitumor effects by destructing the structure of bacteria and tumor cells (Watts et al., 1995; Maness et al., 1999; Liou and Chang, 2012). In contrast, TiO_2_ is considered a stable and safe substance that is used as an additive in food and drugs (Ophus et al., 1979; Skocaj et al., 2011). TiO_2_ coating on biocompatible alloy has the potential to be an ideal technology due to its antibacterial performance by UV irradiation, as well as inherent safety and stability.

Ti6Al4V alloy is a biocompatible material that is resistant to corrosion, therefore it is widely used in orthopaedic implants (Head et al., 1995). However, the elasticity modulus of Ti6Al4V (Young’s modulus: 110 GPa) is higher in comparison to human cortical bone (10–30 GPa) (Long and Rack, 1998). In medical practice, differences of Young’s modulus between femoral implants used in total hip arthroplasty and that of human cortical bone may cause disproportionate stress distribution and lead to pain in the thigh (Glassman et al., 2006). For the resolution of these problems, β-type TiNbSn alloy which has a lower Young’s modulus <50 GPa was developed to reduce the possibility of stress shielding and thigh pain (Miura et al., 2011). β-structured TiNbSn alloy increases its stiffness and Young’s modulus when the alloy is annealed at temperatures above 673 K (Hanada et al., 2014). The results of a clinical trial of a TiNbSn alloy hip prosthesis have been reported, showing that the TiNbSn alloy hip prosthesis was effective in deterring thigh pain and bone atrophy due to stress shielding 3 years after surgery (Chiba et al., 2021). Previous reports have also described that tibial fracture healing in mice and rabbits was enhanced using intramedullary nails made with TiNbSn alloy in comparison to using Ti6Al4V alloy and stainless steel (Fujisawa et al., 2018; Kogure et al., 2019; Mori et al., 2021). To improve the biocompatibility of TiNbSn alloy, it underwent anodic oxidation with acetic acid and sulfuric acid which demonstrated improved osseointegration with hydroxyapatite formation in experimental models (Tanaka et al., 2016; Masahashi et al., 2017; Kunii et al., 2019; Masahashi et al., 2019). Sodium tartrate has been reported to inhibit the surface cracking of anodic oxides. Since high voltage is applied, there is a high possibility of embrittlement of the oxide film, but sodium tartrate is effective in suppressing such embrittlement (Masahashi et al., 2021). A study of anodic oxidation of TiNbSn alloy with sodium tartrate has reported no change in Young’s modulus after anodic oxidation (Hatakeyama et al., 2021). Thus, TiNbSn alloy is a promising biomaterial in the field of orthopaedic prosthesis. Furthermore, anodic oxidation of TiNbSn alloy with sodium tartrate indicated photocatalytic activity with UV irradiation (Masahashi et al., 2021). We conceived the idea that photocatalytic activity could be applied to antibacterial activity.

To date, there are few reports of the antibacterial ability of TiO_2_ by anodic oxidation (Miura et al., 2015). Furthermore, there are no reports on the effect of anodic oxidation with sodium tartrate on the improvement of surface quality and antibacterial activity of β-type titanium alloy with low Young’s modulus. Therefore, the present study aimed to evaluate the antibacterial effect of UV irradiation of anodized TiNbSn alloy prepared in the sodium tartrate electrolyte. Furthermore, we investigated the effect of H_2_O_2_ addition to the electrolyte on antibacterial and photocatalytic activities of anodized TiNbSn alloy for the purpose of accelerating the formation of well-crystallized TiO_2_ and increasing the antibacterial ability.

## Materials and Methods

### Preparation of Anodized TiNbSn Alloy

The composition of TiNbSn alloy used in the present study was Ti-21Nb-2Sn (at%), and was fabricated by thermo-mechanical treatment using extrusion and swaging. The detailed procedure was as previously published (Hanada et al., 2013). TiNbSn alloy plates with dimensions of 25 × 25 × 1 mm, 10 × 20 × 1 mm and 10 × 10 × 1 mm were polished with emery paper (1,500 grit), rinsed in ethanol using an ultrasonic cleaner, and prepared as the anode electrode. Anodic oxidation was performed on the TiNbSn alloy plates for 30 min in 50 mM-sodium tartrate containing 0.7 M-H_2_O_2_ galvanostatically at a constant current density of 50 mA/cm^2^ up to a maximum of 380 V using a DC power supply (PRK 500-3.2, Matsusada Precision, Japan), as previously described (Masahashi et al., 2021). TiNbSn alloy plates anodized with only the 50 mM-sodium tartrate were also prepared to assess the effect of H_2_O_2_ addition. The anodized electrode was rinsed with distilled water, dried at 293 K, and subsequently annealed for 5 h at 723 K in the atmosphere. The anodized TiNbSn alloy with dimensions of 10 × 10 × 1 mm and 10 × 20 × 1 mm were used for the electron spin resonance (ESR) tests and methylene blue (MB) bleaching tests, respectively. The anodized alloy with dimensions of 25 × 25 × 1 mm were used for surface analyses and antibacterial tests.

### Surface Analyses

Surface analyses of the anodized alloy were performed as previously described (Masahashi et al., 2017, 2019; Masahashi et al., 2021). The microstructure of the samples was observed using scanning electron microscopy (SEM; VE-8900, Keyence, Japan), laser microscopy (VK-X 150, Keyence, Japan), and analyses by X-ray Diffraction (XRD; X’Pert diffractometer, PANalytical, Netherlands) with a thin-film geometry arrangement using a 0.5° glancing angle, and a rotating detector was also performed. The upper surface of the samples was analyzed by X-ray photoelectron spectroscopy (XPS) equipped with an electron spectrometer (Kratos AXIS-Ultra DLD, Shimadzu, Japan) with monochromated Al Kα radiation at a base pressure of 3.0 × 10^–7^ Pa. The full width at half maximum intensity of the Ag 3d5/2 peak was 0.73 eV, and the base pressure of the spectrometer was 6.5 × 10^–8^ Pa. The analysis of absorption spectrum was performed using a UV-vis spectrophotometer (V-550, Jasco, Japan).

### Photocatalytic Assessment

For the evaluation of the photocatalytic activity under UV light irradiation (SLUV-4, As-one corporation, Japan), the amount of •OH production was measured by X-band ESR spectrometer (JES-FA-100, JEOL, Japan) using the spin-trapping agent 5,5-dimethel-a-pyrroline-N-oxide (DMPO, Labotech, Japan) as previously described (Iwatsu et al., 2020). UV light irradiation was performed with a wavelength of 365 nm and intensity of 1.0 mW/cm^2^. The photocatalytic activity was also evaluated using MB bleaching tests according to the evaluation method of Japanese Industrial Standards, JIS R 1703-2:2014. The anodized oxide was placed in an optical quartz cell containing 2 ml of 25 mg/L MB aqueous solution until the concentration of MB became constant, as to avoid the effect of MB adsorption on the photocatalytic activity. A UV lamp was used to supply the UV light at a wavelength of 365 nm, and the intensity of the irradiated light was 1.0 mW/cm^2^ at the surface (Masahashi et al., 2009).

### Antibacterial Assays

**FIGURE A1:**
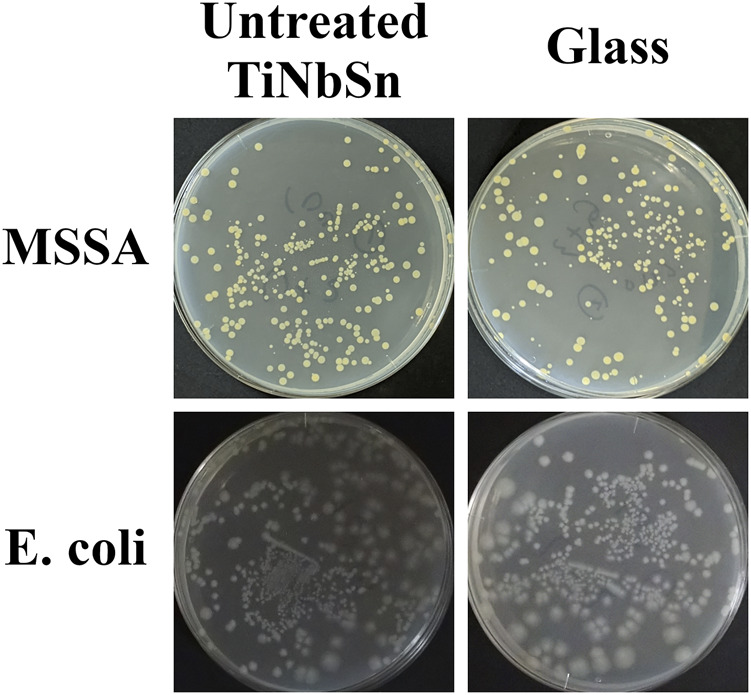
Bacterial culture tests on untreated TiNbSn alloy and glass plate. The photographs show the results of the culture of MSSA and *E. coli* washout solution after antibacterial tests with untreated TiNbSn alloy and glass plate under low-intensity UV light irradiation (0.21 mW/cm^2^) for 8 h. Three independent experiments were conducted. Representative results are shown. *E. coli: Escherichia coli,* MSSA: Methicillin-resistant *Staphylococcus aureus*.

Two different antibacterial tests were performed in the present study. The first antibacterial test was performed according to the evaluation methods of the International organization for standardization, ISO 27447:2019 (Japanese Industrial Standards, JIS R 1702:2012). For the Gram-positive coccus assays, methicillin-sensitive *Staphylococcus aureus* (MSSA; NBRC12732) and methicillin-resistant *Staphylococcus aureus* (MRSA; ATCC43300) were evaluated for their antibacterial activities. For the Gram-negative *bacillus*, *Escherichia coli* (*E. coli*; NBRC3972) was used for the antibacterial assays. Each bacterium was cultured on nutrient agar (Difco nutrient agar, Becton Dickinson, NJ, United States) medium at 35°C for 36–43.5 h. The cultured bacteria were prepared on a 1/500 density of nutrient broth (Nutrient broth, Eiken Chemical, Japan) medium to obtain a bacterial count of 5.3 × 10^6^ cells/mL. This solution was utilized for the antibacterial test. Before antibacterial testing of anodized TiNbSn alloy samples, the antibacterial performance of untreated TiNbSn alloys was explored in comparison with that of glass. Antibacterial tests of MSSA and *E. coli* under low-intensity UV light irradiation demonstrated no antibacterial performance in either untreated TiNbSn alloy or glass (Figure A1). Based on the above results, a glass plate was set as the control for the antibacterial test in this study. Three plates of anodized TiNbSn alloy and three plates of glass as the negative control were used in ISO27447 antibacterial test. Each TiNbSn alloy plate or glass was placed on a plastic net and placed on paper containing 6 ml of sterile water to retain moisture in each Petri dish. The anodized TiNbSn alloy plates and glasses were inoculated with 37.5 μL (2 × 10^5^ cells) of the test bacterial solution, and both the solution and samples adhered with a sterile polyethylene film (VF-10, Kokuyo, Japan). To prevent drying, a 1.1 mm thick glass plate (TEMPAX, Schott, Germany) was placed on top of the Petri dish. The intensity of UV light transmitted through the film and glass plate was 0.21 mW/cm^2^, and the bacteria were incubated with UV light irradiation at a wavelength of 352 nm (FL 20S Bl-B 20W, Nippon Electric Company, Japan) for 8 h at 25°C. For the control group, the bacteria on each sample were cultured in the dark for 8 h at 25°C. After UV irradiation, the samples and film were placed in a plastic bottle containing 20 ml of soybean-casein digest broth with lectin and polysorbate (SCDLP) medium (SCDLP broth, Eiken chemical, Japan), and the test bacterial solution was removed through washing. A volume of 100 μL of the bacterial washout SCDLP medium solution was diluted in 900 μL of saline solution, resulting in a 1/10 dilution of bacterial washout solution. To the nutrient agar medium, 100 µL of bacterial washout solution and 1/10 diluted solution were added and subsequently incubated. The number of viable bacteria was determined by measuring the number of colonies formed with incubation at 35°C after 40–48 h on nutrient agar. The antibacterial activity value (R_L_) of TiNbSn alloy and the effect of UV light irradiation (△R) had been calculated from the following equation:

$${\text{R}}_{\text{L}}=\log10\left({\text{G}}_{\text{L}}/{\text{T}}_{\text{L}}\right)$$

$$\text{Δ}\text{R}=\log10\left({\text{G}}_{\text{L}}/{\text{T}}_{\text{L}}\right)-\log10\left({\text{G}}_{\text{D}}/{\text{T}}_{\text{D}}\right)$$

T_L_: The average number of viable bacteria on three pieces of TiNbSn alloy plates after 8 h of UV irradiation.

G_L_: The average number of viable bacteria on three pieces of glass after 8 h of UV irradiation.

T_D_: The average number of viable bacteria on three pieces of TiNbSn alloy plates after 8 h of storage in the dark.

G_D_: The average number of viable bacteria on three pieces of glass after 8 h of storage in the dark.

For both cases, the antibacterial activity was defined to be more than 2.0 in following ISO 27447 (JIS R 1702). Where no viable bacteria were observed in the anodized TiNbSn alloy, the viable bacteria count (T_L_) was recorded as 10.

A time decay antibacterial assay was conducted for MRSA which is considered as one of the most problematic bacteria clinically. This was conducted by partially modifying ISO27447 by increasing the UV intensity to 1.0 mW/cm^2^ and shortening the irradiation time from 8 h to less than 3 h. The logarithmic decrease in the number of viable bacteria remaining on anodized TiNbSn alloy prepared in the electrolyte with or without H_2_O_2,_ and on glass at 1, 2, and 3 h from time point zero was compared.

### Statistical Analyses

Statistical analyses were performed using JMP, Version 15 (SAS, NC, United States). Statistical significance between the values of •OH in the ESR method was determined using one-way analysis of variance (ANOVA) and post hoc analysis using Tukey-Kramer test. The correlation between time and the production of •OH was evaluated using Spearman’s rank correlation test. For the time decay antibacterial tests, significant differences in logarithmic decrease in the number of viable bacteria at each time point were determined using one-way ANOVA and post hoc analysis using Steel-Dwass test. Values of <0.05 were considered statistically significant.

## Results

### Surface Analyses

**FIGURE 1:**
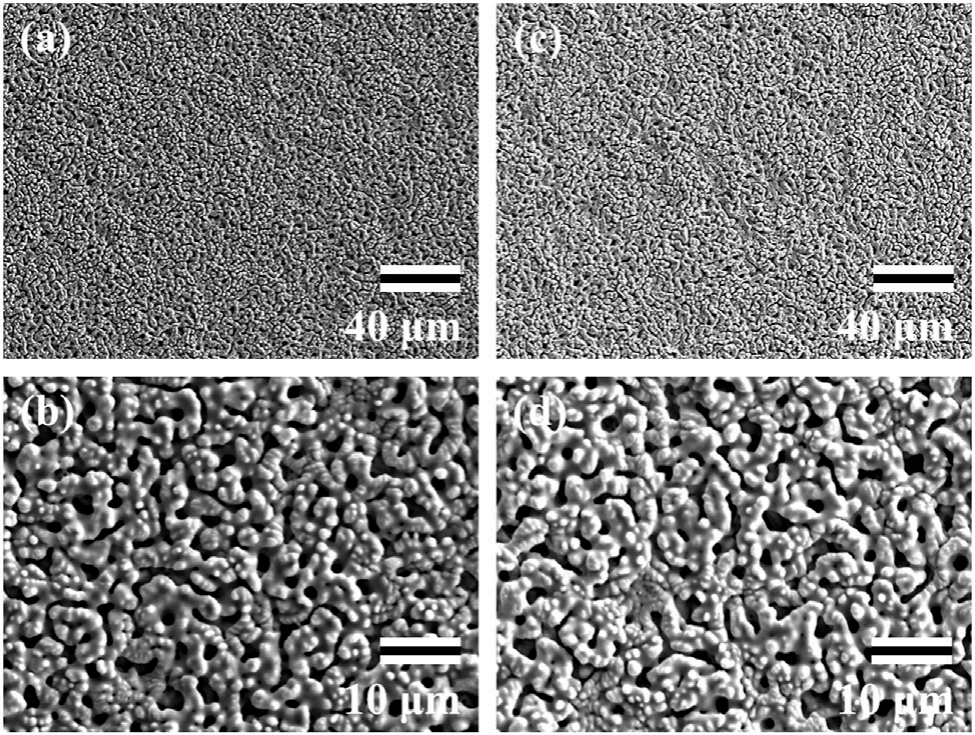
SEM images of the anodic oxides prepared in a sodium tartrate electrolyte. a and b indicate anodic oxides with H_2_O_2_, c and d indicate anodic oxides without H_2_O_2_. Three independent experiments were conducted. Representative results are shown. Scale bar is 40.0 μm in the lower magnification images **(A,C)** and 10.0 μm in the higher magnification images **(B,D)**. SEM: Scanning electron microscope.

**FIGURE 2:**
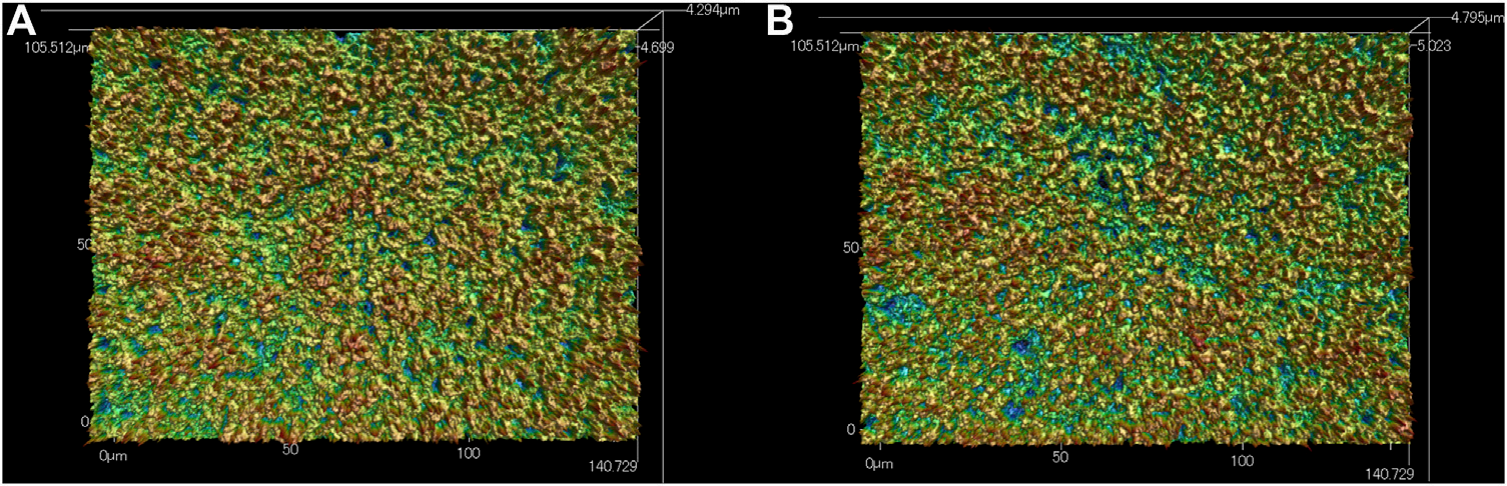
Laser Microscopic images of the anodic oxides prepared in a sodium tartrate electrolyte. **(A)** indicates anodic oxides with H_2_O_2_, **(B)** indicates anodic oxides without H_2_O_2_. Three independent experiments were conducted. Representative results are shown.

The SEM data for surface analyses of the anodized TiNbSn alloy are shown in Figure 1. The anodized alloy had uniform porous microstructures with fine micropores. There was no difference in the formation of porous microstructure between anodized oxide prepared in the electrolyte with and without H_2_O_2_. The laser microscopy analyses (*n* = 3) are shown in Figure 2. The mean values for surface roughness of the anodized alloy with and without H_2_O_2_ were 0.495 and 0.470 µm, respectively. The measurements of the surface area ratio, which are the ratios of the measured surface area to the projected area were 2.8 and 2.8%, respectively. There were no differences in the surface roughness and surface area ratio regardless of the electrolyte composition.

**FIGURE 3:**
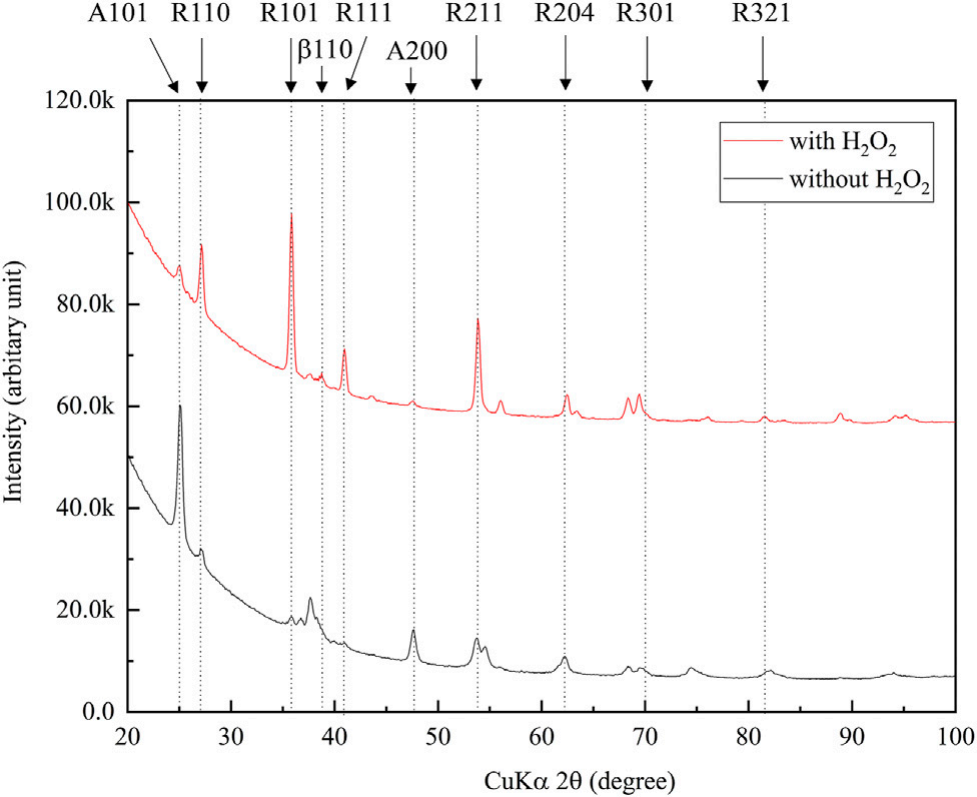
XRD narrow scan profiles of the anodic oxides prepared in the sodium tartrate electrolyte with and without H_2_O_2_. The peaks of rutile-structured TiO_2_ were accelerated in anodic oxide with H_2_O_2_. The sample size is *n* = 1 in each group. XRD: X-ray Diffraction.

**FIGURE 4:**
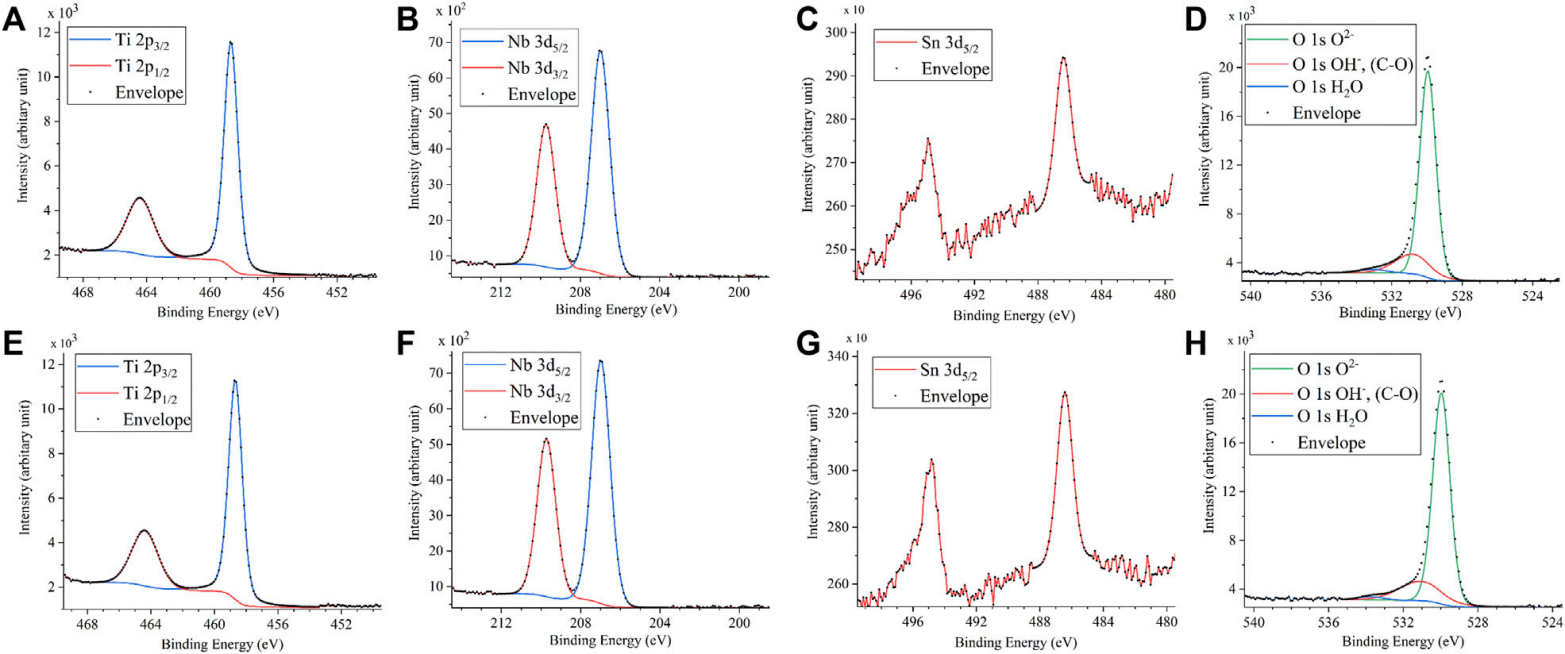
XPS spectra of **(A)** Ti 2p, **(B)** Nb, **(C)** Sn, and **(D)** O 1s on the anodic oxide with and **(E)** Ti 2p, **(F)** Nb, **(G)** Sn and **(H)** O 1s on the anodic oxide without H_2_O_2_. The sample size is *n* = 1 in each group. XPS: X-ray photoelectron spectroscopy.

**FIGURE 5:**
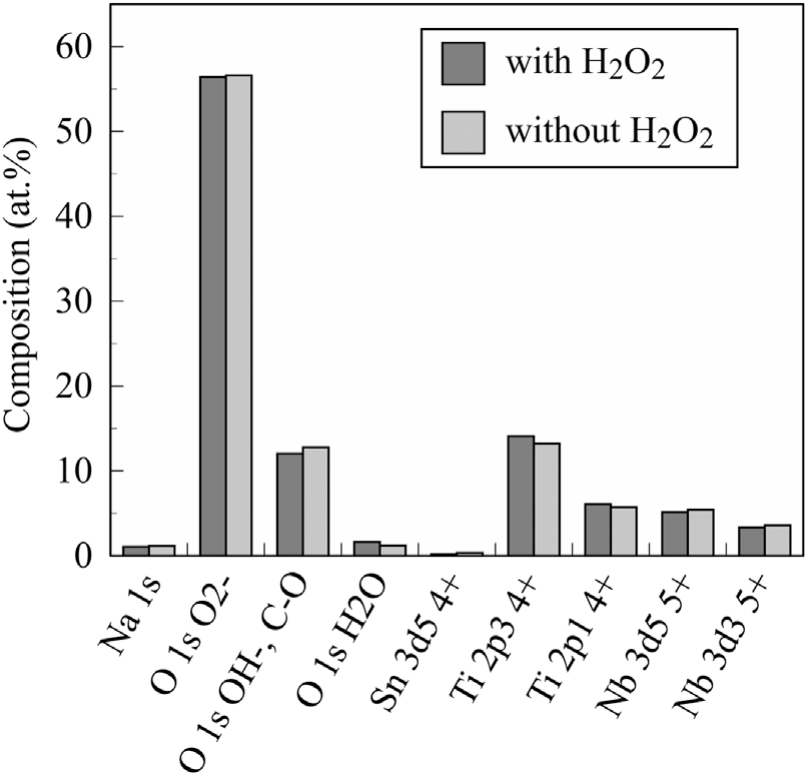
The chemical composition of investigated anodic oxides on the TiNbSn alloy calculated by semi-quantitative analysis using the XPS spectra. The sample size is *n* = 1 in each group. XPS: X-ray photoelectron spectroscopy.

**FIGURE 6:**
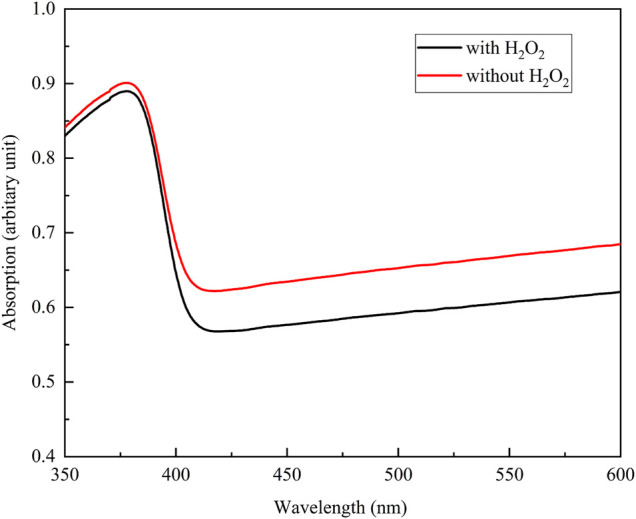
The absorption spectra of the anodic oxide prepared in the sodium tartrate electrolyte with and without H_2_O_2_. The sample size is *n* = 1 in each group.

Figure 3 shows the XRD profiles of the anodic oxide of TiNbSn alloy prepared in the electrolyte with and without H_2_O_2_. Anatase- and rutile-structured TiO_2_ were detected in both anodized TiNbSn alloy, however, the peaks of rutile-structured TiO_2_ were accelerated in the anodized TiNbSn alloy prepared in the electrolyte with H_2_O_2_. The XPS spectra of the anodic oxides had peaks for the Ti, Nb, Sn, O, S, and C, and a weak N peak. Peaks corresponding to C and N originated from contamination during sample preparation and air exposure. The anodic oxide on TiNbSn alloy was composed of TiO_2_, Nb_2_O_5_, and SnO or SnO_2_, as was previously described (Tanaka et al., 2016; Kunii et al., 2019). The Ti 2p revealed that the surface was completely composed of titanium oxide for the oxides prepared in the electrolyte prepared in the electrolyte with and without H_2_O_2_. The symmetrical shape of the Ti 2p spectrum suggested that reduced Ti^3+^ ions were not present in the oxides. The O 1s peak at approximately 529.9 eV was ascribed to oxygen in TiO_2_, and shoulder peaks at a higher binding energy side than the main peak was originated from H_2_O. The Ti 2p3/2, Nb 3d5/2, and Sn 3d5/2 spectra had a peak corresponding to the binding energy of approximately 458.8, 207.3, and 486.6 eV, respectively, and they could be assigned as TiO_2_, Nb_2_O_5_, and SnO or SnO_2_ from the literature (Figure 4) (Charstain, 1992). Semi-quantitative analysis utilizing the XPS spectra concluded that any distinct difference between the oxides prepared in the electrolyte with and without H_2_O_2_ was not observed (Figure 5). Assuming that the oxides were composed of TiO_2_, Nb_2_O_5_, and SnO_2_, the fraction of oxides was calculated from the atomic fraction of the metal. The fraction of constituent oxides was estimated as 62 and 65% of TiO_2_, 37 and 34% of Nb_2_O_5_, and 1.1 and 0.6% of SnO_2,_ for the anodic oxide prepared in the electrolyte with and without H_2_O_2_, respectively. It was concluded that both the chemical composition and constituent oxide fraction was almost similar among both oxides. The absorption spectra of the anodized TiNbSn alloy, which were prepared in a sodium tartrate electrolyte (a) with and (b) without H_2_O_2_ was evaluated (Figure 6). Absorbances of the anodic oxides decrease with increasing wavelength, which is a typical spectrum of semiconductors. The sharp decrease in absorbance with increasing wavelength was a little more pronounced in (a) than in (b), but there was no significant difference between the spectra of (a) and (b). These results suggest that crystallized TiO_2_ was formed on TiNbSn alloy and H_2_O_2_ addition to the electrolyte did not accelerate the maturation of the crystallized structure of TiO_2_.

### Photocatalytic Assessment

**FIGURE 7:**
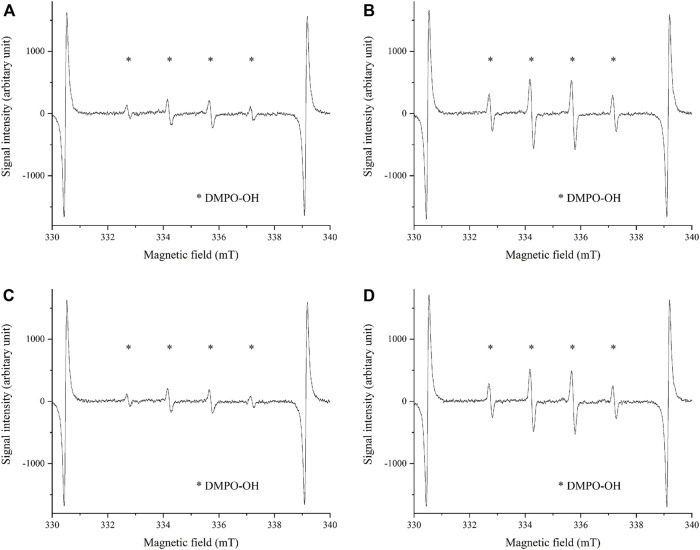
The •OH spectrum by ESR method on anodic oxides prepared in the sodium tartrate electrolyte with and without H_2_O_2_. The peaks of •OH are shown in both anodic oxides by ESR methods using DMPO. The sample size is *n* = 3 in each group. **(A)** UV irradiation on anodic oxide with H_2_O_2_ for 5 min **(B)** UV irradiation on anodic oxide with H_2_O_2_ for 15 min **(C)** UV irradiation on anodic oxide without H_2_O_2_ for 5 min **(D)** UV irradiation on anodic oxide without H_2_O_2_ for 15 min. ESR: Electron spin Resonance, DMPO: 5,5-dimethel-a-pyrroline-N-oxide.

**FIGURE 8:**
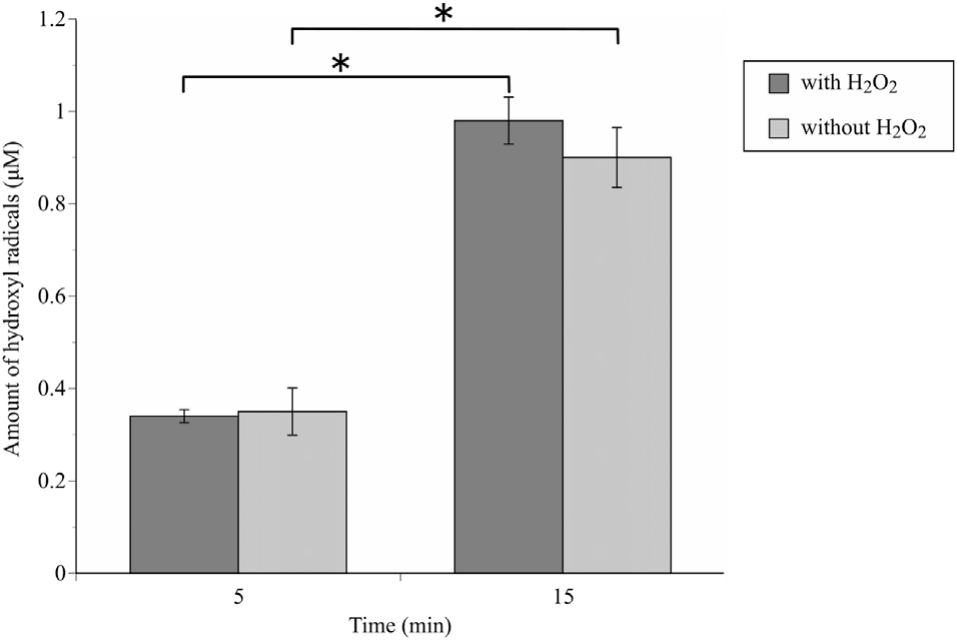
The comparison of the amount of generated •OH radicals by ESR methods between anodic oxides in the sodium tartrate electrolyte with and without H_2_O_2_. The sample size is *n* = 3 in each group. The one-way ANOVA post hoc by Tukey-Kramer test was used for statistical evaluation. *: *p* < 0.05 ESR: Electron spin Resonance, ANOVA: analysis of variance.

**FIGURE 9:**
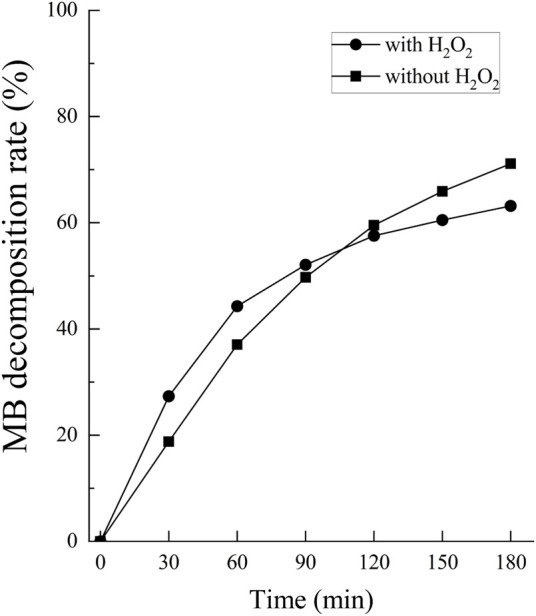
The comparison of the degradation rate of MB between anodic oxides in the sodium tartrate electrolyte with and without H_2_O_2_. The sample size is *n* = 1 in each group. MB: Methylene blue.

The •OH spectrum on anodized TiNbSn alloy prepared in the electrolyte with and without H_2_O_2_ by ESR method is shown in Figure 7 (*n* = 3). The quantitative assessment of the amounts of •OH is shown in Figure 8. There was no significant difference in the amounts of •OH between the anodized TiO_2_ prepared in the electrolyte with or without H_2_O_2_ at both the 5 and 15 min in time point (*p* = 0.817 and *p* = 0.369, respectively). There was a strong correlation between the amount of •OH and UV irradiation time in both the anodized TiO_2_ prepared in the electrolyte with and without H_2_O_2_ (*r =* 0.878, *p* = 0.021 with the addition of H_2_O_2_; and *r* = 0.891, *p* = 0.017 without the addition of H_2_O_2_). The reduction ratio of the MB bleaching test is plotted in Figure 9 and there was no difference in the reduction ratio between the anodic oxides prepared in the electrolyte with or without H_2_O_2_.

### Antibacterial Test

**FIGURE 10:**
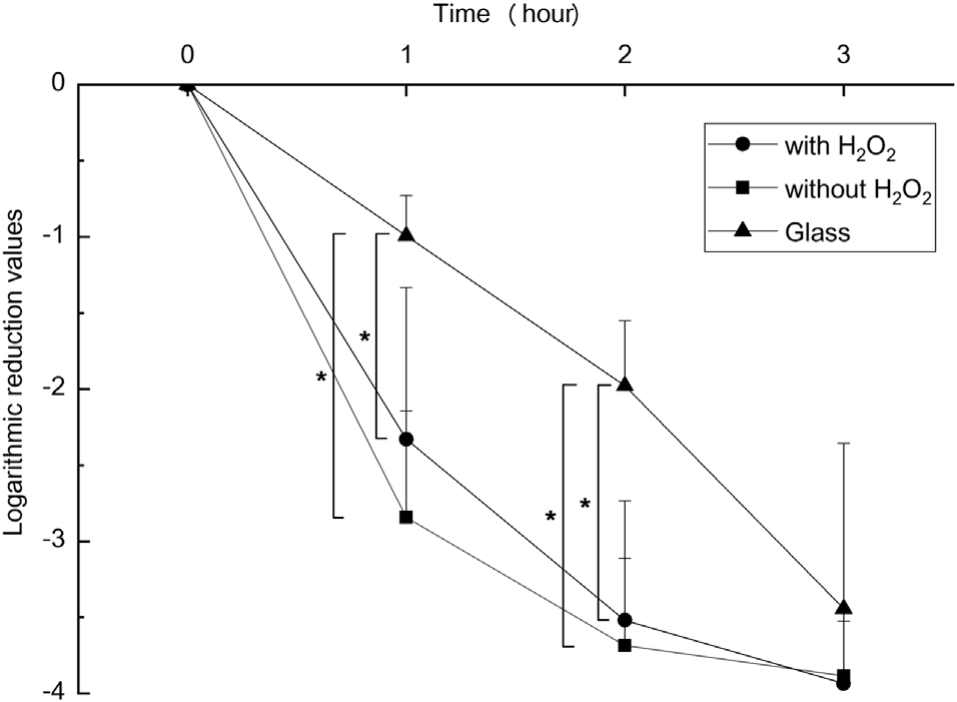
The comparison of logarithmic reduction values in each specimen. The sample size is *n* = 5 in each group. The one-way ANOVA post hoc by Steel-Dwass test was used for statistical evaluation. *: *p* < 0.05 ANOVA: analysis of variance.

An antibacterial test was conducted in duplicate for each bacterial species according to the ISO27447 antibacterial test. The antibacterial activity values of the anodized TiNbSn alloy plates with 0.21 mW/cm^2^ of UV light irradiation are detailed in Table 1. The antibacterial activity values with the UV light irradiation were more than 2.0 for all the bacterial species regardless of the addition of H_2_O_2_ to the electrolyte. These results demonstrate that the photocatalytic activity of the anodized TiNbSn alloy under UV irradiation had a robust antibacterial effect. The logarithmic reduction values obtained in the antibacterial test were calculated using glass, anodized TiNbSn alloy prepared in the electrolyte with or without H_2_O_2_ under 1.0 mW/cm^2^ of UV light irradiation (*n* = 5 (Figure 10). The logarithmic reduction values of the anodized TiNbSn alloy were significantly larger than those of glass at 1 and 2 h. The logarithmic reduction values of MRSA after 1-h with UV light irradiation were significantly increased in both anodized TiNbSn alloy prepared in the electrolyte with and without H_2_O_2_, in comparison to the glass (*p* = 0.033 and *p* = 0.033 with and without addition of H_2_O_2_ respectively). Similarly, after 2-h of UV light irradiation, the logarithmic reduction values were significantly increased in both anodized TiNbSn alloys compared to those of the glasses (*p* = 0.033 and *p* = 0.033 with and without addition of H_2_O_2_ to the electrolyte, respectively). In contrast, there was no significant differences after 3-h of UV light irradiation between all the groups. Comparing the logarithmic reduction values between the anodized TiNbSn alloys prepared in the electrolyte with and without H_2_O_2_, there were no significant differences in the two groups after 1- and 2-h UV light irradiation.

**TABLE 1:** The assessment of antibacterial values and effects of photocatalysis according to the ISO 27447 test.

	Bacteria	Antibacterial Values	Effect of photocatalysis
With H_2_O_2_	MSSA	3.90, 3.82	3.21, 3.64
	MRSA	2.53, 3.69	2.14, 2.63
	*E. coli*	2.99, 4.20	3.01, 4.29
Without H_2_O_2_	MSSA	3.90, 3.82	3.41, 2.98
	MRSA	3.35, 3.69	3.03, 2.74
	*E. coli*	4.21, 4.20	3.58, 4.33

The assessment was performed in duplicate per assay and repeated in triplicate. Representative data is shown.

MSSA, methicillin-sensitive Staphylococcus Aureus; MRSA, methicillin-resistant Staphylococcus Aureus; E. coli, Escherichia coli.

## Discussion

### Overview

In this study, the antibacterial activity of anodized TiNbSn alloy, prepared in a sodium tartrate electrolyte with and without H_2_O_2_, was demonstrated. The addition of H_2_O_2_ to the electrolyte did not alter the porous microstructures and the absorption transition of the anodic oxide prepared in the H_2_O_2_ free electrolyte. Furthermore, it did not change the photocatalytic activity and the amount of generated •OH. Although it is inferred that the addition of H_2_O_2_ accelerated the formation of well-crystallized TiO_2_, there was no significant difference in the photocatalytic and antibacterial performances between the two anodic oxides prepared in the electrolyte with and without H_2_O_2_.

### Achievement of TiO_2_ With the Formation of Highly Crystallized and Porous Oxides Regardless of H_2_O_2_ Addition

**FIGURE A2:**
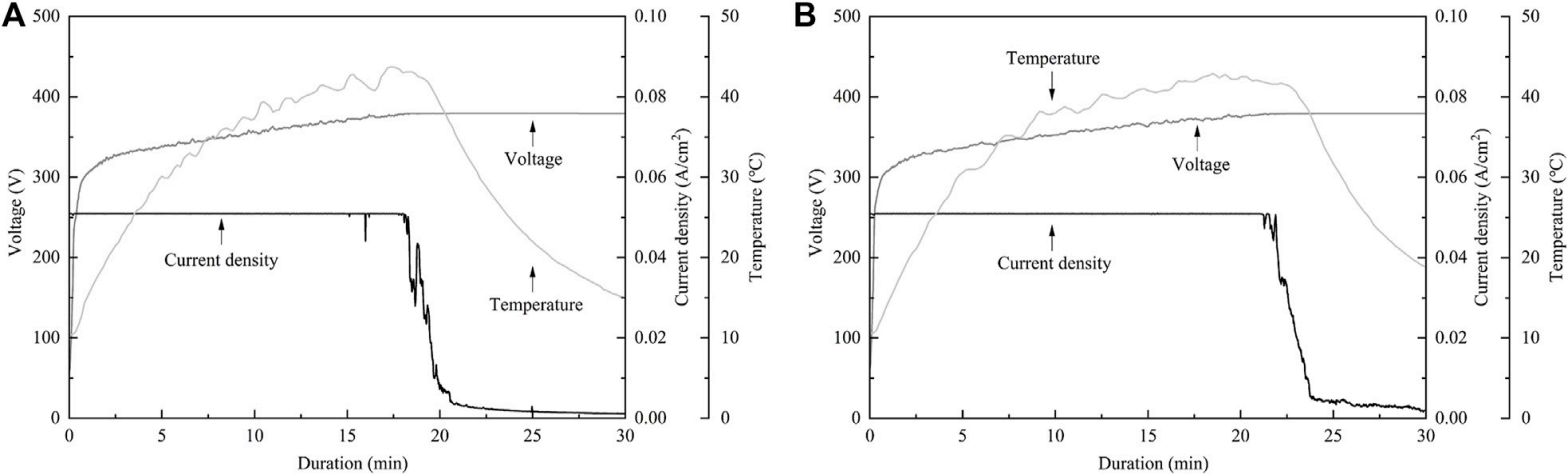
The electrochemical behavior monitored by the electrolysis Curve. **(A)** The electrolysis curve in anodic oxide with H_2_O_2_, **(B)** The electrolysis curve in anodic oxide without H_2_O_2_. The sample size is *n* = 1 in each group.

In the present study, SEM and laser microscopy demonstrated that anodic oxide on TiNbSn alloy prepared in a sodium tartrate electrolyte had a fine porous microstructure regardless of H_2_O_2_ addition to the electrolyte. Previous studies have reported that dielectric breakdown leads to porous microstructure formation on the anodic oxide prepared in an electrolyte of sulfuric acid aqueous solution (Diamanti and Pedeferri, 2007; Park et al., 2007). In a recent study, anodic oxide prepared in the electrolyte of sodium tartrate with applying a constant voltage of 500 V developed a porous microstructure regardless of the addition of H_2_O_2_ to the electrolyte (Masahashi et al., 2021). Those results were consistent with the results of the present study. The authors considered that the higher the anodic oxidation voltage is applied, the more frequent breakdown occurs, resulting in the formation of highly crystallized and porous oxides. Since H_2_O_2_ is a strong oxidizing agent, we added H_2_O_2_ to sodium tartrate, a weak acid, in the expectation that it would accelerate the anodic oxidation reaction. We consider that the promotion of anodic oxidation reactions leads to an increase in the crystallinity of TiO_2_. Actually, the addition of H_2_O_2_ to the electrolyte accelerated anodic oxidation reaction, which is verified by the galvanostatically controlled duration during anodization (Figure A2). In the present set-up for anodization, the galvanostatic controlling mode changes to the potentiostatic controlling mode when the electrode voltage arrived at the set voltage of 380 V. This electrochemical behavior was monitored by the electrolysis curve, and the curve revealed that the addition of H_2_O_2_ reduced the galvanostatically controlled duration (Figure A2). High voltage induces dielectric breakdown accompanied by spark discharge at the surface of the TiNbSn electrode, which promotes crystallization of anodic oxide owing to self-heating (Masahashi et al., 2021). The galvanostatic period is shorter with the addition of H_2_O_2_ than without the addition of H_2_O_2_. Voltage also increases in the short term, and self-heating also progresses earlier, which is presumed to be advantageous for the maturation of the anodic oxide. In contrast, reduction of the galvanostatically controlled duration retards the crystallization of anodic oxide, which deteriorates the photocatalytic activity due to the high density of lattice defects. Low crystallinity increases the recombination probability of photogenerated charges, resulting in low photocatalytic activity. It is considered that the effect of H_2_O_2_ addition to the electrolyte on photocatalytic activities was weakened by the above two contradictory effects.

### Increased Ratio of Rutile-Structured TiO_2_ by H_2_O_2_ Addition did Not Affect Photocatalytic Activity

The XRD analysis demonstrated that the addition of H_2_O_2_ to the electrolyte increased the ratio of rutile-structured TiO_2_. It has been reported that anatase-structured TiO_2_ had higher bandgap energy than rutile-structured TiO_2,_ and ROS generated from the anatase structure had a longer lifetime than rutile (Xu et al., 2011; Zhang et al., 2014; Hu et al., 2018). Therefore, anatase-structured TiO_2_ has been reported to have higher photocatalytic activity than rutile-structured TiO_2_. From those previous studies, anatase-structured TiO_2_ exhibits an advantage in photocatalytic activity and antibacterial performance, however, there were no significant differences between anodic oxides prepared in the electrolyte with and without H_2_O_2_. Thus, there was no difference in photocatalytic activity and antibacterial performance between TiO_2_ after anodic oxidation with rutile and anatase structures. The authors considered this is ascribed to the density of lattice defects acting as recombination sites, and further studies are required to elucidate.

The previous study reported that the addition of H_2_O_2_ treatment demonstrated no apparent improvements in the evaluation of radical production by ESR examination (Masahashi et al., 2021). Those results were consistent with the present study, and ESR examination demonstrated that the amounts of •OH significantly increased depending on the duration of the UV irradiation regardless of H_2_O_2_ addition. The degradation rate of MB also increased depending on the duration of the UV irradiation regardless of H_2_O_2_ addition to the electrolyte, suggesting that the photocatalytic activity varied with illumination time.

### TiNbSn Alloy With Anodic Oxidation in a Sodium Tartrate had the Antibacterial Activity With or Without H_2_O_2_ Addition

The ISO27447 antibacterial test demonstrated that the anodic oxide on TiNbSn alloy prepared in a sodium tartrate with and without H_2_O_2,_ had antibacterial activity against MSSA, MRSA, and *E. coli,* under the low intensity of UV irradiation. These results suggest that the photocatalytic performance of anodic oxide on TiNbSn alloy was suitable for the sterilization of pathological bacteria, regardless of drug resistance, and that this antibacterial performance may be suitable for the clinical setting. The authors considered that this anodic oxidation technique could potentially contribute to the problem of refractory bacterial infection including vancomycin-resistant bacteria (Wright, 2007). While there are side effects to antibacterial drugs, the TiO_2_ surface is considered safe due to the anodic oxide strongly adhesive on the substrate, and TiO_2_ being biologically inert (Ophus et al., 1979; Punjataewakupt et al., 2019). The antibacterial effect of anodic oxide was considered suitable in the terms of safety.

The time decay antibacterial test was performed to identify whether the antibacterial activity could be achieved in a shorter time using higher intensity UV irradiation than that within the ISO27447 test. The anodic oxide on TiNbSn alloy prepared in the electrolyte with and without H_2_O_2_ significantly reduced MRSA. The authors considered that three-hour UV irradiation directly killed MRSA, however irradiation below 2 hours demonstrated antibacterial activity by photocatalysis of anodic oxide on TiNbSn alloy. It has already been previously reported that anodic oxide has antibacterial activity (Yeniyol et al., 2015), however, results in our study showed much higher antibacterial activity compared to the previous report. In the results of ESR tests, •OH radicals were generated in 5 min of UV irradiation, suggesting a possible antibacterial effect in a short-time of UV irradiation. Increased intensity of UV irradiation and larger surface of anodized samples can be expected to increase the amount of •OH radicals generated even with short-time UV irradiation. We would like to examine whether stable anodic oxidation is possible for large specimens in future research. This study is the first step toward imparting antibacterial performance by anodic oxidation for titanium alloys in biological applications. In the application of TiNbSn alloy to hip and knee prosthesis, UV irradiation on hip and knee prosthesis anodized with sodium tartrate during surgery for surgical site infection may be useful for killing multidrug-resistant bacteria, thus contributing to infection control and prosthesis preservation. With the development of the technique of anodic oxidation on TiNbSn alloy, the development of biomedical materials that exhibit antibacterial activity by UV irradiation is expected.

## Conclusion

The photocatalytic activity of the anodic oxide of TiNbSn alloy prepared in a sodium tartrate with or without H_2_O_2_ was demonstrated. The anodized oxide exhibited a porous microstructure, and well crystallized anatase and rutile-structured TiO_2_ regardless of H_2_O_2_ addition to the electrolyte. The abundant generation of •OH and photocatalytic activity under UV irradiation was confirmed in the anodic oxide on TiNbSn alloy prepared in the electrolyte with and without H_2_O_2_, and there was no difference between the two types of anodic oxides. In the antibacterial test, no significant difference was detected between anodic oxide on TiNbSn alloy with and without H_2_O_2_, and both anodic oxides on TiNbSn alloy indicated a robust antibacterial activity. Anodic oxide on TiNbSn alloy may be a promising biomaterial with low Young’s modulus and antibacterial performance.

## Data Availability

The original contributions presented in the study are included in the article/Supplementary Material, further inquiries can be directed to the corresponding author.

## Abbreviations

ANOVA, Analysis of variance; DMPO, 5,5-dimethel-a-pyrroline-N-oxide; E. coli, *Escherichia coli;* ESR, Electron spin resonance; H_2_O_2_, Hydrogen peroxide; ISO, International organization for standardization; JIS, Japanese Industrial Standards; MB, Methylene blue; MRSA, Methicillin-resistant *Staphylococcus aureus;* MSSA, Methicillin-sensitive *Staphylococcus aureus;* •OH, Hydroxyl radicals; ROS, Reactive oxygen radicals; SCDLP, Soybean-casein digest broth with lectin and polysorbate; SEM, Scanning electron microscopy; TiO_2_, Titanium dioxide; UV, Ultraviolet; XPS, X-ray photoelectron spectroscopy; XRD, X-ray diffraction.
